# Telepractice Delivery of Caregiver Coaching for Parents of School-Aged Children with Autism Spectrum Disorder in Taiwan: A Pilot Study

**DOI:** 10.3390/bs15020118

**Published:** 2025-01-24

**Authors:** Ching-Yi Liao, Yuet-Yee Yumi Chan

**Affiliations:** Department of Special Education, Social Emotional Education and Development Center, National Taiwan Normal University, 162, Section 1, Heping E. Rd., Taipei City 106, Taiwan

**Keywords:** autism spectrum disorder, parent coaching, behavior intervention, communication intervention, telepractice

## Abstract

Parental involvement is essential in interventions aimed at enhancing communication outcomes for children with autism spectrum disorder (ASD). Research has shown that parents can effectively implement evidence-based strategies following professional coaching. However, there is a notable gap in research on the procedures of parent coaching provided to families of children with ASD in Taiwan. This study aims to evaluate a protocol for distance-delivered parent coaching focused on the implementation of evidence-based strategies for parents of children with ASD. This study employed a multiple-probe design across participants to assess both parent implementation of intervention strategies and the communication outcomes of the children involved. The results demonstrated that the online parent coaching program effectively increased parents’ use of evidence-based intervention strategies, which corresponded to measurable improvements in the target communication behaviors of children with ASD. Also, this study highlighted potential challenges, such as the influence of children’s challenging behaviors on the intensity and effectiveness of parent coaching. These findings contribute to the clinical significance of distance caregiver coaching as a possible approach to supporting families of children with ASD, particularly in underserved areas. The necessity of tailoring service intensity and incorporating culturally responsive practices for the diverse needs of families of children with ASD and effective intervention implementation is discussed in this study.

## 1. Telepractice Delivery of Caregiver Coaching for Parents of School-Aged Children with Autism Spectrum Disorder in Taiwan: A Pilot Study

Autism spectrum disorder (ASD) is a neurodevelopmental disorder characterized by persistent difficulties with social communication, restricted interests, and repetitive behaviors (APA, 2022). The U.S. Centers for Disease Control and Prevention (CDC) reports that approximately 163,000 children were diagnosed with ASD, representing an average of 1 in 44 children (Autism Speaks, 2021; National Institute of Mental Health, 2024). In Taiwan, statistics from the Ministry of Health and Welfare show that the confirmed cases of ASD surged to 19,078 in 2020, nearly 90 times the number in the past 20 years. This rapid upward trend in diagnoses suggests that ASD students are projected to constitute approximately 14.64% of the student population in public education by 2022 (Taiwan Ministry of Health and Welfare, 2022). These statistics highlight the increasing prevalence of ASD, raising significant concerns among healthcare professionals and educators.

ASD is characterized by a spectrum of symptoms that vary significantly in severity and manifestation, with early signs often emerging before the age of three, including communication difficulties such as limited speech or challenges in understanding social cues, as well as restricted interests and repetitive behaviors like intense focus on specific topics or routines (APA, 2022). Many children with ASD face significant challenges in developing social interaction and communication skills to the extent that they may struggle to develop adequate verbal and communication abilities to meet their daily needs (Curcio & Paccia-Cooper, 1981; van Santen et al., 2013). Notably, boys are nearly five times more likely to be diagnosed with ASD than girls, a disparity that may be attributed to differences in how symptoms present across genders (APA, 2022; National Institute of Mental Health, 2024). These characteristics create significant challenges for children with ASD in establishing and maintaining positive social interactions and limit opportunities for communication with others in their daily life, thereby affecting their life skills and academic performance. Furthermore, these challenges may extend into adulthood, impacting employment opportunities (Boyd et al., 2011; Dogoe et al., 2010; Eilers & Hayes, 2015; van Santen et al., 2013). Therefore, intervention strategies designed to address communication skills in children with ASD are crucial for their development.

Since social communication difficulties are a hallmark of ASD, it is important for therapeutic services to involve all communication partners in the child’s daily environments. Implementing intervention strategies in natural settings (e.g., home, school, and community) ensures that children with ASD have more opportunities to practice and generalize the communication skills they learn, leading to more meaningful and lasting improvements (Kasari et al., 2015). However, access to professional services remains an important issue in ASD education and treatment. Many families of children with ASD have the gap problem between their needs and the ability to obtain professional services, which might negatively affect a children’s development, achievement, and quality of life (Antezana et al., 2017; Roddy & O’Neill, 2019). Therefore, developing a family-centered approach is an important option for family caregivers to actively participate in their children’s intervention process (Ingersoll & Wainer, 2013).

Parent involvement means professionals in educational and therapeutic settings work closely with family caregivers (e.g., parents or siblings) to support a child’s development. An important method of such engagement is parent coaching, whereby professionals provide caregivers with the required knowledge, parenting skills, and support. Parents then apply these strategies across different natural settings to improve and maintain their children’s communication skills (Brown & Woods, 2015; Garbacz et al., 2016; Johnson et al., 2016; Schertz et al., 2017; Schreibman et al., 2015; Wacker et al., 2005). Behavioral Skills Training (BST; Parsons et al., 2012) is a well-established, evidence-based, and structured caregiver training model, which includes steps to describe, demonstrate, practice, and provide feedback on target skills until proficiency is achieved, thereby supporting development in children with ASD. Parent training has a positive impact on family well-being, with empowered and confident parents reducing stress and increasing satisfaction (Brookman-Frazee, 2004; Keen et al., 2010). Working with professionals allows parents to become active partners who share responsibility in their child’s education, improving the outcomes for children with ASD.

Advances in technology have promoted the development of telepractice, which employs telecommunications and network technologies to provide remote screening, assessment, intervention, consultation, and education in children with complex communication needs (Christopoulou et al., 2022; Peterson, 2024). Previous research has shown that telepractice effectively improves parents’ implementation of intervention strategies and communication skills in children with ASD, while also helping to reach families in rural areas and bridge the gap between available services and those in need (Benson et al., 2018; Meadan et al., 2022; Simacek et al., 2017; Vismara et al., 2013). Through receiving professional training and support via distance practice, parents can effectively implement evidence-based strategies in natural settings, thereby improving their children’s communication opportunities within daily experiences (Hong et al., 2016; Kaiser & Roberts, 2013; Kaiser et al., 2000; Law et al., 2004; Meadan et al., 2016; Schreibman et al., 2015). In rural areas of Taiwan, insufficient government subsidies to support professionals in directly delivering family support services have led to limited resources for therapeutic interventions and parenting consultations. This has resulted in a restricted scope of service coverage and inadequate alignment with local cultural needs, further hindering the accessibility and availability of these essential services (Lin, 2013). Although a previous review has demonstrated that telepractice can enhance verbal communication abilities in children with complex communication needs, limited research and no formal regulations in Taiwan currently govern the implementation of telepractice in this field (L. Y. Yang et al., 2024).

In Asia, increasing attention has been focused on the role and the involvement of parents in different outcomes of children with ASD and other developmental disabilities over the past few decades (Acar et al., 2021; Cheng et al., 2023). Research has shown that parent coaching can support parents to effectively decrease undesired communication behaviors (e.g., screaming) and increase verbal and nonverbal communication skills (e.g., joint attention and the use of communication aids; Cheng et al., 2023; Liang et al., 2020). By obtaining more knowledge about intervention strategies for their children with ASD, parents can employ these techniques across a variety of natural settings to continuously strengthen and promote their children’s communication skills (Chu et al., 2015; Gao & Drani, 2024; H.-L. Lee, 2015; Liou et al., 2005; Nevill et al., 2018; Y. Yang & Ge, 2015). However, previous relevant research has generally focused on younger children and parent–child interactions rather than the details of parent coaching (Flippin et al., 2010; Ganz et al., 2012; Gerow et al., 2018; Nevill et al., 2018), as well as a lack of considerations of the cultural contexts in Asia (Gao & Drani, 2024; Syed et al., 2022). Therefore, further research is urgently needed to develop and evaluate culturally appropriate telepractice protocols of parent coaching for Asian caregivers of children with ASD.

The purpose of this study was to evaluate the effectiveness of a telepractice parent coaching program to improve parents’ implementation of intervention strategies and the communication outcomes of children with ASD. The research questions are as follows:

1. Is there a functional relationship between the telepractice parent coaching program and the accuracy of parents’ implementation of intervention strategies?
2. How effective is parent-implemented intervention strategies in improving the communication skills of children with ASD?
3. How do parents evaluate the social validity of the telepractice parent coaching program?

## 2. Method

### 2.1. Participants

This study recruited three children with ASD, aged 8, 9, and 10 years, along with their parents. Inclusion criteria included that they were diagnosed with ASD through medical or educational evaluations, exhibited moderate to moderate-mild communication difficulties, demonstrated at or above average cognitive abilities (the relevant information and/or documentation provided by parents), and utilized one or more communication modes (e.g., verbal speech, gestures, or communication aids). Also, the children did not have severe genetic or metabolic disorders, nor present significant sensory or physical impairments that would substantially affect their communication abilities. To better understand the children’s communication behaviors, this study employs the Chinese version of the *Vineland Adaptive Behavior Scales™, Third Edition* (*Vineland-3*; Sparrow et al., 2016) as a supportive tool, which is completed by the parents. This assessment evaluates the children’s social and communication abilities.

Three children participated in the current study. Child A was a 9-year-old boy diagnosed with level 2 ASD, requiring substantial support. He communicated using simple sentences in both Chinese and English, although his language skills were delayed compared to his same-age peers. Child A preferred to use an iPad for communication, exhibited avoidance of eye contact, and interacted primarily through narrow special interests. The parents reported that he had difficulties in emotional regulation, showing challenging behaviors (e.g., removing clothing during class, taking off his shoes, throwing books, or lying on the floor) to avoid entry into the classroom. He had received early intervention services, applied behavior analysis (ABA), occupational therapy, speech therapy, and physical therapy since kindergarten age.

Child B was an 8-year-old boy diagnosed with level 1 ASD, requiring support. His communication mode was speech, which was characterized by repetitive speech and a preference of gestures. Also, he used a limited set of sentence structures and words. Due to Child B’s stereotyped behavior and difficulties in understanding others, his misunderstandings often led to outbursts like yelling and crying. Before participating in the current study, he had received early intervention services, occupational therapy, speech therapy, and physical therapy since the age of four.

Child C was a 10-year-old male diagnosed with Asperger syndrome, classified under level 1 ASD, requiring support. Despite possessing fluent and age-appropriate verbal skills, he experienced difficulties in social interactions. Child C exhibited lower behavioral and emotional control, often freezing when anxious and misinterpreting others’ intentions. He displayed repetitive (e.g., taking others’ belongings without permission) and aggressive behaviors (e.g., throwing objects, shouting, and hitting his mother with slippers when feeling angry or rejected). He had received special education, occupational therapy, and physical therapy since kindergarten age.

All three participating parents are mothers of the children involved, with ages ranging from 46 to 49 years. Their educational backgrounds vary, encompassing degrees from bachelor’s to doctoral levels. Only one parent had previously attended a single parenting education seminar, while the other two parents had no prior experience with ASD-related activities. For further details, please refer to Table 1.

### 2.2. Settings

The study was conducted in the home of each participant dyad and took place during naturally occurring routines. Parent coaching sessions were held approximately once per week via WebEx. For Child A, the target routine involved sharing experiences about school life during dinner. For Child B, the target routine focused on discussing the events of the day in the living room. For Child C, the target routine centered on sharing daily experiences after school. Parents were instructed to interact with their children and record videos based on these daily routines.

### 2.3. Dependent Measure

The dependent variables were both the implementation of intervention strategies by parents and the communication behaviors of children. The implementation of intervention strategies was defined as the frequency with which parents accurately apply the following strategies: (1) Environmental Arrangement: parents adjust materials, activities, and communication partners within natural settings to create increased opportunities for children to practice communication, as well as use a reinforcer to enhance children’s motivation to communicate; (2) Modeling: parents demonstrate target communication behaviors through verbal expressions, gestures, physical actions, or communication aids for children to imitate; (3) Prompting: parents provide cues or hints (i.e., verbal prompts, gestures, or physical assistance) designed to encourage the child to engage in target communication behaviors; (4) Expanding: parents encourage children to utilize more complex or varied forms of communication like longer or more complete sentences. The independent variable is the distance-delivered parent coaching program.

Target communication behaviors for each child participant were identified through their communication needs and modes via assessments and interviews. The operational definitions of the target behaviors include the following: (1) Spontaneous Comments: spontaneous comments for Child A and Child B were defined as the use of verbal communication, gestures, or communication aids to communicate relevant information to the conversation context and then pauses for approximately 2 to 3 s for the parent to respond; (2) Asking Questions: Asking questions for Child C was defined as the use of verbal communication, gestures, or communication aids to formulate appropriate questions that are relevant to the conversation context. Also, the children pause for about 2 to 3 s for the parent’s answer.

The observational recording method utilized is partial-interval recording. This method involves recording the occurrence of target behaviors at 10 s intervals within three-minute video segments. The frequency of target behavior occurrences is captured; if the target behavior occurs at any point during the interval, it is recorded. The data collection form includes the operational definitions for each target behavior along with their corresponding codes. When a target behavior is observed, the relevant code is marked on the form. The coach reviewed the videos uploaded by the parents across the phases to collect the necessary data.

### 2.4. Design and Procedures

This study employed a multiple-probe design across participants, a specific type of single-subject experimental design, to investigate the effectiveness of a parent coaching program in enhancing two key outcomes: (1) the implementation of intervention strategies by parents and (2) the communication skills of children with ASD. The research procedure consisted of three distinct phases: baseline, intervention, and maintenance. Generalization data were collected across the baseline, intervention, and maintenance phases to evaluate the extent to which the intervention strategies learned were applied in various contexts. A total of 8 sessions across both the intervention and maintenance phases were provided to each parent participant.

#### 2.4.1. Baseline Phase

The coach conducted semi-structured interviews with each parent participant for the information regarding the child’s current communication skills, communication modes, environmental cues for initiating and terminating communication, preferred items or activities, challenging behavior(s), and the parents’ prioritization of target communication behavior(s). The interview questions also included family background and routines, the understanding of ASD among family members, the parents’ educational background, and any ASD-related training parents have received. Based on the results from *Vineland-3* and the interviews, the parents and the coach identified the target communication behaviors for each child and developed intervention plans.

Following the evaluation and interviews, the coach collaborated with each parent to establish a communication context that aligns with the family’s daily routines and supports the child’s communication development. Parents recorded interactions based on this context. In the baseline phase, parents interacted with their children and captured three-minute video recordings of these interactions, which were subsequently uploaded to Microsoft OneDrive. At this stage, no guidance or feedback on performance was provided. Given the challenges associated with recording in natural settings and the typically brief nature of social interactions in children with ASD, a three-minute video duration was deemed both feasible and acceptable for most parents. In accordance with the experimental design and family routines, five recordings were collected from Dyad A, four from Dyad B, and six from Dyad C in the baseline phase.

#### 2.4.2. Intervention Phase

The intervention phase commenced when the baseline data indicated that the dyad required an intervention. In accordance with the principles of the multiple-probe design across participants, the timing for the second and third dyads to initiate the intervention phase depended on the stability and effectiveness of the data obtained from the previous dyad. Stability is defined as the range of values for the last three data points falling between 0% and 30%.

1. First Stage: Foundational Knowledge Course

The two-hour foundational knowledge online course, which was adapted from the content of the prior research (Liao et al., 2022; Ura et al., 2021), aims to provide foundational knowledge on behavioral interventions (i.e., environmental arrangement, demonstration, prompting, and expanding) and multimodal communication techniques (e.g., verbal communication, gestures, and communication aids) to all participating parents. The contents covered definitions, appropriate timing, situational contexts, and procedural steps for each intervention strategy through the methodology of explanations, demonstrations, case analyses, and interactive quizzes to ensure comprehension. All parents must complete this foundational knowledge course before proceeding to individual coaching sessions.

2. Second Stage: Individual Parent Coaching

The six-week individual parent coaching sessions were conducted via WebEx weekly to provide tailored guidance on the application of behavioral interventions and multimodal communication techniques, ensuring accuracy in implementation and fostering the child’s communication skills. The procedures and contents were developed based on the previous studies (Liao et al., 2022; Meadan et al., 2016; Ura et al., 2021) and grounded in the BST (Parsons et al., 2012), which include the following steps:

(1) Oral Explanation: target skills explained in detail by the coach;
(2) Written Materials: supporting documents shared via WebEx and Microsoft OneDrive;
(3) Demonstration: live demonstration of intervention strategies by the coach;
(4) Role-Playing: parents practice intervention strategies under guided scenarios;
(5) Immediate Feedback: real-time feedback provided during role-playing sessions;
(6) Repetition for Mastery: steps 4 and 5 repeated until the parent demonstrates mastery of the skills;
(7) Q&A Discussion: open discussion to address parents’ questions and concerns.

Following each weekly coaching session, parents applied the learned intervention strategies with their child in natural settings. Interaction sessions were recorded (three-minute videos) and uploaded weekly to Microsoft OneDrive for analysis and feedback. The coach reviewed the recorded parent–child interactions to collect data and assess the accuracy of implementation. Feedback was provided to improve the implementation of skills and address challenges in subsequent sessions.

#### 2.4.3. Maintenance Phase

Upon completion of the intervention phase, the parents recorded a three-minute video of their interactions with their children every two weeks, demonstrating the use of the intervention strategies and then uploaded the videos to Microsoft OneDrive for review. The coach analyzed the videos for data collection and provided verbal and written feedback to parents for the maintenance of target strategies and behaviors. Each parent participated in two coaching sessions for ongoing support of the skills learned to ensure the sustainability of the intervention effects and facilitate continued progress in the parent’s and children’s outcomes.

#### 2.4.4. Generalization Phase

The generalization phase for each child participant was conducted based on each of their preferences and family routines. Child A and Child C watched a preferred video (e.g., videos about trains and models) and subsequently discussed the content with their parents. Child B’s generalization phase included discussing daily life and experiences in the child’s bedroom.

### 2.5. Data Analysis

In the current study, Tau-U was employed as a non-parametric measure (Vannest et al., 2016) to assess both parents’ implementation of the strategies and the child’s target communication behaviors. Tau-U was selected for data analysis due to its widespread use in single-case experimental design (SCED) studies to assess effect sizes by evaluating both within-phase trends and across-phase differences. It accounts for baseline trends, corrects for undesirable baseline trends via statistical adjustments, and calculates non-overlap between the baseline and intervention phases (J. B. Lee & Cherney, 2018; Parker et al., 2011), thereby making Tau-U a robust and sensitive tool for detecting intervention effects. Compared to other metrics like the percentage of non-overlapping data, Tau-U was chosen for its conceptual foundation and demonstrated predictability, as seen in previous studies (Brossart et al., 2018). Tau-U scores range from −1.0 to 1.0, where positive scores indicate an increasing trend in behavior and negative scores indicate a decreasing trend. The established benchmarks were used for the interpretation of Tau-U: scores from 0.93 to 1.00 for large effects, from 0.80 to 0.92 for medium effects, from 0.65 to 0.79 for small effects, and below 0.64 for very small effects (Ganz et al., 2017).

### 2.6. Interobserver Agreement (IOA)

Two observers, both first- and second-year master’s students in the Special Education program with experience working with individuals with ASD, evaluated interobserver agreement (IOA). Prior to independently coding the data, the observers received training in data recording from the first author, a board-certified behavior analyst at the doctoral level, and were required to achieve a minimum agreement score of 90%. Retraining was provided if their agreement percentage fell below this threshold. To assess IOA, a second observer independently coded at least 20% of the videos, which were randomly selected from each phase and each dyad. The data coded by the second observer were then compared to the primary observer’s codes. All IOA coding procedures were supervised by the first author. IOA was calculated by dividing the number of agreements by the total number of agreements plus disagreements and then multiplying the result by 100 to obtain a percentage. The results indicated that the average IOA was 94%.

### 2.7. Procedural Integrity

These two observers also evaluated the procedural integrity of the parent coaching sessions via a procedural integrity checklist. Before their independent coding, both observers completed training in procedural integrity coding and achieved over 90% accuracy in practice activities. At least 20% of the videos was randomly selected and coded from each phase and each dyad. The results of procedural integrity revealed an average score of 100%.

### 2.8. Social Validity

A parent coaching feedback survey was employed to evaluate parents’ satisfaction with and acceptability of the intervention of the parent coaching. The autism-specific parenting self-efficacy scale (Kurzrok et al., 2021), the parent satisfaction survey (Washburn, 2012), the parent coaching short survey (Liao et al., 2022), and the treatment evaluation inventory short form (TEI-SF; Kelley et al., 1989) were adapted to develop the survey. Following the individual parent coaching sessions, the online survey was distributed to parents for anonymous completion. The study calculated scores and averages for each item on a five-point Likert scale based on participant responses and consolidated qualitative feedback from open-ended questions.

## 3. Results

**Figure 1:**
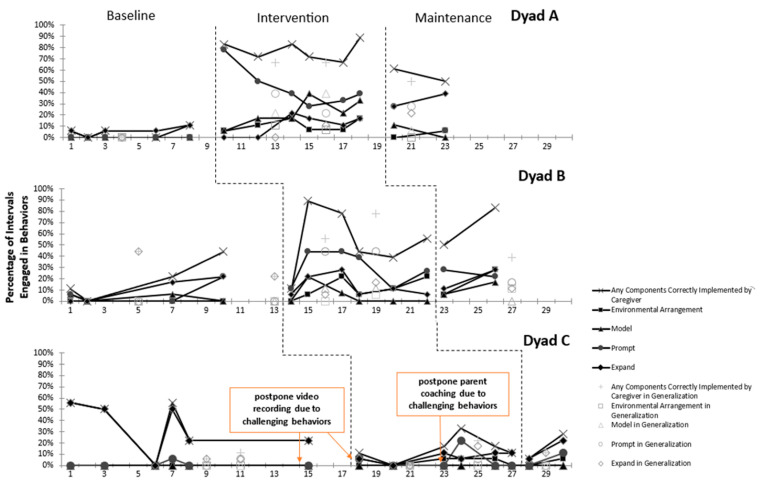
Parents’ implementation of intervention strategies across phases.

**Figure 2:**
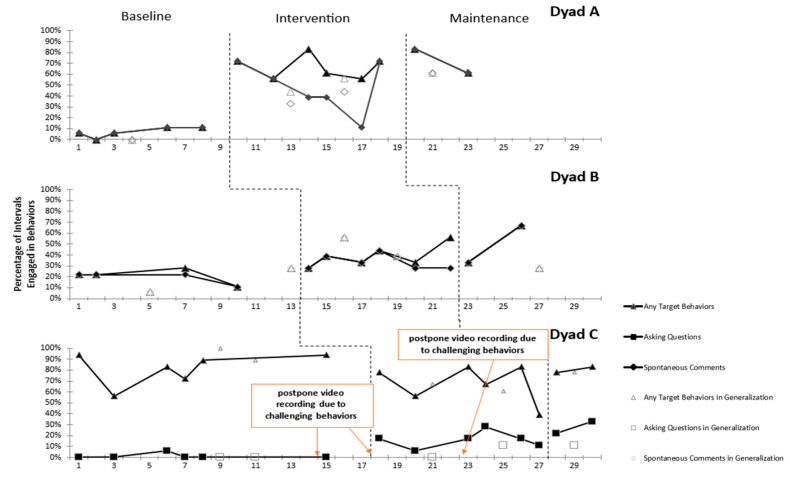
Performance of children’s target communication behaviors across phases.

This study employed visual analysis (see Figure 1 and Figure 2) and Tau-U effect size analysis (see Table 2 and Table 3) to evaluate the effectiveness of the parent coaching program on parents’ implementation of intervention strategies and the communication behaviors of their children. Parents applied the strategies learned from parent coaching in natural settings, focusing on interactions and conversations with their children. However, Child C exhibited unexpected challenging behaviors in the baseline phase, which continued into the intervention and maintenance phases. Although these challenging behaviors did not fall within the study’s exclusion criteria and, for ethical reasons, the child was not excluded from participation, the parent had to prioritize addressing these behaviors. This focus on managing the child’s challenging behaviors resulted in delays in the parent’s coaching sessions. Consequently, the visual analysis and effect size results varied across the dyads, reflecting the impact of these challenges on the overall outcomes of the study.

### 3.1. Parent Implementation of Intervention Components

#### 3.1.1. Dyad A

A visual analysis of Parent A revealed an increasing trend and higher levels in the use of all strategies learned during the parent coaching compared to the baseline period. The range of strategy use in the intervention and maintenance phases was 67% to 89%. Generalization data for Parent A showed lower levels of strategy use in the baseline phase, but both the trend and levels increased in the intervention and maintenance periods.

Regarding specific strategies, the environmental arrangement strategy exhibited a gradual increase in usage (Figure 1). Although no environmental arrangement strategies were employed in the baseline phase, an increasing trend with slight variations emerged in the intervention phase and continued into the maintenance phase. For modeling, Parent A did not utilize demonstration strategies in the baseline phase; however, there was a stable increase in its use in the intervention phase, with a noticeable upward trend in the later part of this phase. In terms of prompting strategies, Parent A initially did not use prompting in the baseline phase. However, by the beginning of the intervention phase, they quickly mastered prompting techniques, resulting in a rapid increase in usage levels ranging from 28% to 78%. Despite a slight decline in prompting frequency due to practicing other strategies within the limited video time, the overall trend remained higher than in the baseline phase. Lastly, regarding expansion, Parent A used it only twice by the end of the baseline phase. Following the intervention phase, there was a gradual increase in its use, influenced by the interactive environment and dialog content with the child, extending into the maintenance phase.

The effect size analysis shows that Parent A exhibited a large overall effect size (ES = 0.93), indicating a substantial impact on the use of all strategies following parent coaching. This includes a large effect size for environmental arrangement, modeling, and prompting strategies (ES = 1.00). However, since children’s target communication behaviors had not reached mastery during parent coaching, Parent A did not have opportunities to expand the child’s communication behaviors. The effect size for expanding strategies was very small (ES = 0.20).

#### 3.1.2. Dyad B

In Parent B, the use of all learned strategies exhibited a significant increase in the intervention phase compared to the baseline phase, with usage percentages ranging from 11% to 89% in both the intervention and maintenance phases. Although there was a slight decrease in strategy use following the third and fourth coaching sessions in the intervention phase, the overall trend remained upward and continued into the maintenance phase.

Regarding the environmental arrangement strategy, parents initially did not employ this approach in the baseline phase. However, an increasing trend in its use was observed in the intervention phase, albeit with some variability, and this upward trend persisted into the maintenance phase. In terms of modeling, parents demonstrated very low usage in the baseline phase, with percentages ranging from 0% to 6%. Following the second and third coaching sessions in the intervention phase, the use of modeling increased to 22% and 6%, respectively. This elevated level of modeling continued into the maintenance phase, with percentages ranging from 6% to 17%. Prompting was another strategy where parents demonstrated rapid mastery in the intervention phase, showing increased usage and higher levels compared to the baseline phase. However, a decrease to 11% was noted following the fifth coaching session; this was attributed to contextual factors and limited opportunities for using the strategy. After the sixth coaching session, the use of prompting strategies increased and stabilized throughout the maintenance phase. The use of expanding strategies was initially limited, with some increase observed in the intervention phase. Despite a decline after the fourth coaching session, an upward trend was reestablished in the maintenance phase.

The effect size analysis revealed a medium overall effect size (ES = 0.50) for Parent B. The effect size for environmental arrangement was medium (ES = 0.83), while modeling exhibited a very small effect size (ES = 0.08). Prompting demonstrated a medium effect size (ES = 0.79), and expanding showed a very small effect size (ES = 0.08). These findings indicate that, like Parent A, the target communication behaviors for the children did not reach a mastery level, resulting in a minimal effect for the expanding strategy.

#### 3.1.3. Dyad C

In Parent C, severe challenging behaviors observed in the final baseline phase and the first and third parent coaching sessions in the intervention phase led to delays in filming and coaching sessions. The parents needed time to address their child’s behavior and seek medical attention. As a result, the overall trend in the use of all learned strategies by the parents showed a decline compared to the baseline phase, with baseline percentages ranging from 0% to 56% and intervention and maintenance phase percentages ranging from 0% to 33%. Generalization data for Parent C indicated low levels of strategy use in the baseline phase, with a slight increase in the intervention and maintenance phases (see Figure 1).

Regarding environmental arrangement, Parent C demonstrated low levels of use, ranging from 0% to 6%, and did not use the modeling strategy at all. For prompting, Parent C used the prompting strategy once in the baseline phase and showed a slight increase and variability in the intervention phase. However, there was a downward trend after the fifth coaching session. Concerning expanding, parents initially used a higher amount of this strategy in the baseline phase but showed a rapid decline, which continued into both the intervention and maintenance phases, where it remained stable at lower levels.

Effect size calculations revealed a negative small overall effect size (ES = −0.44) for Parent C. Specifically, the effect size for environmental arrangement was medium (ES = 0.50); modeling showed no effect; prompting had a very small effect size (ES = 0.17), and expanding demonstrated a negative very small effect size (ES = −0.50). These findings suggest that the severe problem behaviors exhibited by Child C significantly impacted the parents’ ability to effectively implement the learned strategies, leading to a negative effect on the overall outcomes.

### 3.2. Child Communicative Behaviors

#### 3.2.1. Dyad A

In the baseline phase, Child A exhibited low levels of all communication behaviors. However, upon the commencement of the intervention phase, there was a noticeable increase in both the trend and level of the child’s communication behaviors, with the range in the intervention and maintenance phases being between 56% and 83%. The generalization data for Child A also indicated that while communication behaviors were at a low level in the baseline phase, both the trend and level increased in the intervention and maintenance phases.

Specifically, regarding Child A’s target communication behavior of spontaneous comments, the visual analysis indicated a slight increase in the baseline phase, with a range of only 6% to 11%. In the intervention phase, both the trend and level of the child’s spontaneous comments increased significantly, with the range extending from 11% to 72%. Although there was a decrease to 11% in the target behavior’s percentage following the fifth parent coaching session, attributed to the child asking numerous questions in the recorded video, an upward trend resumed in the latter stages of the intervention phase and continued into the maintenance phase (see Figure 2).

Further analysis of the effect size revealed that the parent coaching program showed a moderate effect on the child’s target communication behavior (see Table 3). Specifically, the effect size for the child’s target behavior of spontaneous comments was moderate (ES = 0.73), and the effect size for all communication behaviors was also moderate (ES = 0.80). These findings suggested that the parent coaching was effective in increasing the child’s ability to produce spontaneous comments and engage in appropriate communication behaviors.

#### 3.2.2. Dyad B

Compared to the baseline phase, Child B exhibited higher levels and an increasing trend in all communication behaviors in the intervention phase, which continued into the maintenance phase, with a range of 28% to 67% in these periods. The generalization data similarly indicated that communication behaviors were at a low level in the baseline phase; however, both the trend and level increased in the intervention and maintenance phases. Specifically, regarding the target communication behavior of spontaneous comments, Child B demonstrated low levels of performance in this area in the baseline phase. However, there was a notable increase in both the trend and level in the intervention phase, which persisted into the maintenance phase, with a range of 33% to 67% (see Figure 2).

The effect size analysis results presented in Table 3 further indicated that Child B exhibited a very large effect size for the target communication behavior (ES = 1), and all communication behaviors in the natural setting also demonstrated a very large effect size (ES = 1). These findings suggest that the interventions implemented were highly effective in enhancing the child’s ability to produce spontaneous comments, indicating a significant improvement in overall communication skills.

#### 3.2.3. Dyad C

Following the baseline assessment, Child C had already mastered the communication behavior of spontaneous comments. Consequently, the target communication behavior was elevated to a more advanced communication skill of asking questions. In terms of all communication behaviors, Child C exhibited a slight downward trend compared to the baseline phase, where performance levels ranged from 56% to 94%, with levels in the intervention and maintenance phases ranging from 39% to 83%.

Regarding the specific target communication behavior of asking questions, Child C demonstrated low levels in the baseline phase, with a range of 0% to 6%. However, in the intervention and maintenance phases, there was a noticeable increasing trend and higher levels of performance, with a range of 6% to 33%. The generalization data indicated that the level of the target communication behavior showed a slight increase in the intervention phase, which continued into the maintenance phase (see Figure 2).

The effect size analysis showed that after the parent received parent coaching, Child C achieved a very large effect size (ES = 1) for the target communication behavior of asking questions. However, when calculating the effect size for all communication behaviors in the natural setting, Child C demonstrated a negative moderate effect size (ES = −0.64). This outcome can be attributed to the child’s already proficient ability to produce spontaneous comments in the baseline phase. In the intervention phase, the focus on developing the target behavior of asking questions within a limited time frame in a three-minute video reduced opportunities for the child to demonstrate spontaneous comments.

### 3.3. Social Validity

This study utilized a parent coaching feedback survey to evaluate parents’ satisfaction and gather feedback regarding the coaching. Upon calculating scores and averages for each survey question, the overall average score was 4.86, indicating that parents rated their responses as either “5 Very Satisfied” or “4 Satisfied” for all questions. Furthermore, qualitative feedback from the open-ended questions showed that parents appreciated the detailed explanations and demonstrations provided by the coach, which facilitated their understanding of the material. Also, parent participants mentioned that they valued the online coaching sessions, which analyzed the videos they recorded. These procedures allowed them to receive feedback and directly adjusted their implementation, thereby guiding their child to achieve the communication goals. Furthermore, the case examples provided by the coach were helpful in addressing specific challenges they encountered in parenting. For future suggestions, the parent participants suggested that the parent coaching program should include additional interventions and assessments with the children after the maintenance phase to ensure that communication outcomes have been effectively improved.

## 4. Discussion

The results of the current study were consistent with previous studies, which demonstrated that telepractice parent coaching effectively improved the knowledge and accurately implemented evidence-based intervention strategies among parents of children with ASD (Benson et al., 2018; Meadan et al., 2022; Simacek et al., 2017; Vismara et al., 2013). The results of the current study showed an increase in parents’ implementation of intervention strategies and children’s targeted communication behaviors. The effect size for the target communication behaviors of Child A and Child B showed a large effect size, indicating that children with ASD can make progress in their target communication behaviors when parents accurately employ the intervention strategies learned in the parent coaching program. These findings suggested the importance of providing evidence-based interventions to families of children with ASD via parent coaching. Equipping parents with the necessary skills and strategies can lead to significant improvements in children’s communication outcomes, even in a telepractice format. Also, the discrepancy between the visual analysis and effect size results highlights the need for multiple measures to assess the effectiveness of the parent coaching program accurately.

Although the visual analysis did not reveal significant changes in Child C’s communication behaviors, this result can be attributed to the simultaneous use of different communication targets (i.e., spontaneous comments and asking questions) by the children in the three-minute natural setting videos. Furthermore, challenging behaviors might significantly affect the consistency of parent coaching sessions, as well as the timing and strategies parents practice when implementing intervention strategies at home. For example, Child C began medical treatment due to behavioral issues toward the end of the baseline period. Consequently, Parent C had to postponed some parent coaching sessions in the intervention and maintenance phases. The child’s challenging behaviors frequently resulted in delays in both participating in parent coaching sessions and recording videos. Furthermore, the videos recorded also reflected the parent’s need to first address the child’s challenging behavior, which limited opportunities for practicing appropriate communication skills. These observations highlight the complexities involved in parent coaching and in the implementation of intervention strategies across natural settings. Considering the family’s living and working environments in Taiwan and behavioral characteristics of children with ASD, flexibility in service delivery in timing and method is crucial to support parents’ completion of all coaching tasks. This also aligns with the findings of previous research in Taiwan (Lin, 2013). Future research and practice in the related field might prioritize the adaptation of parent coaching content and delivery methods to improve the effectiveness of the parent coaching program and support the unique needs of families of children with ASD.

Furthermore, Taiwan’s cultural uniqueness like family dynamics, educational systems, and access to resources may influence how distance coaching intervention and support can be implemented. The process of parent coaching also revealed that the unique living and working environments in Asia, along with the diverse cultures and lifestyles within families, significantly contribute to the challenges faced by caregivers of children with ASD. In this study, although a few fathers expressed interest during the recruitment process, all three parent participants were mothers, consistent with the participant characteristics observed in prior studies (Hu et al., 2017; Rankin et al., 2019). Throughout the parent coaching, mothers frequently reported elevated levels of stress related to raising their children, a lack of knowledge regarding appropriate resources to support their children with ASD and other family members, and concerns about their children’s learning progress and future, which were also observed in previous studies (Brookman-Frazee, 2004; Keen et al., 2010). Access to resources and support from family members were commonly highlighted concerns among participating parents. There is a prevalent misconception that families of children with ASD have sufficient access to resources; however, the adequacy and appropriateness of these resources are often questionable, particularly when considering the family’s geographical location and cultural background. Professionals may consider these factors when working with individuals with ASD and their families in future.

During parent coaching sessions, the parent participants also shared their emotional and psychological distress. The responses from these parents echo the findings of previous research where caregivers of children with ASD often experience higher levels of stress, emotional and psychological distress, and disruptions to family life, which might lead to social isolation and further exacerbate the challenges faced by these families (Brookman-Frazee, 2004; Keen et al., 2010). Given these complexities, it is crucial for professionals to understand the specific needs of families in different contexts and to adapt their support accordingly. Based on these findings, improving resource accessibility and providing tailored coaching approaches can better support and further improve the quality of life for both caregivers and children with ASD.

There are some limitations in the current study, as well as recommendations for future research. (1) Regarding sample size, as a pilot study, one of the key limitations is the small sample size which limits the generalizability and external validity of the results. Future research should include larger sample sizes to improve the generalizability of findings. (2) Regarding methods of parent coaching, the current study solely relied on Webex for delivering parent coaching. Future research should explore the effectiveness of alternative methods, such as bug-in-ear coaching (Hamberger et al., 2022; Ottley, 2016) or virtual reality (VR; Abdeen & Albiladi, 2022; Simmons et al., 2023), to promote online support for caregivers of children with ASD. (3) Regarding research design, the current study employed a single-case experimental design (SCED), which is suitable for small sample sizes, but it limits the generalizability of the results to larger populations. Although a multiple-probe design across participants was implemented to replicate intervention effects, individual variations among dyads might influence the results. For example, the presence of challenging behaviors in children with ASD added complexity to the intervention process. Future research should consider different research designs, clearer criteria, individual factors (e.g., severity of ASD), and more specific intervention adjustments to minimized uncontrolled variability. (4) Regarding video recording duration, the three-minute video probes used for measuring communication behaviors were intentionally kept short to accommodate limited tolerance for change and to minimize frustration for children with ASD (Garretson et al., 1990; Goldstein et al., 2008; Hodgson et al., 2017). While this duration was appropriate given the children’s needs, future research should investigate alternative methods or extend the observation duration for more comprehensive analyses of parent–child interactions. (5) Regarding data analysis, the current study used only visual analysis and Tau-U for data analysis, as well as the thresholds for interpreting Tau-U values informed by the related study. For a more robust justification of these thresholds, future research should apply more comprehensive approaches for data interpretation (e.g., comparisons with other analytical metrics) to ensure the reliability of the results. (6) Regarding maintenance and generalization, the long-term effectiveness of the coaching was not sufficiently addressed, although maintenance and generalization data were analyzed. Future research might include longer-term follow-up observations and evaluations to better understand the sustainability of parent coaching. (7) Regarding the cultural context, a deeper analysis of how different cultural contexts influence the implementation of distance coaching is required, although the study highlights the effectiveness of distance parent coaching in Taiwan. Future research should adapt interventions to consider cultural differences and include diverse populations to ensure that the findings can be generalized to more contexts and populations.

## Figures and Tables

**Table 1:** Participant Information.

	DYAD A	DYAD B	DYAD C
		Parent Participants	
ID	Parent A	Parent B	Parent C
Relationship with the Child	Mother	Mother	Mother
Age	46	49	49
Educational Background	Bachelor’s Degree	Master’s Degree	Doctoral Degree
Experience with Autism-Related Activities	None	Parenting Education Seminars	None
		Child Participants	
ID	Child A	Child B	Child C
Age	9	8	10
Gender	Male	Male	Male
Severity of ASD	Level 2	Level 1	Level 1
Communication Mode	Speech and iPad	Speech and Gesture	Speech

**Table 2:** Effect sizes for the parent coaching program on parents’ use of intervention strategies.

Intervention Strategy	Dyad	Tau-U	*p*-Value	LL CI 90%	UL CI 90%
Environment Arrangement	Parent A	1.00	0.01 *	0.40	1.00
	Parent B	0.83	0.03 *	0.19	1.00
	Parent C	0.50	0.15	−0.07	1.00
Modeling	Parent A	1.00	0.01 *	0.40	1.00
	Parent B	0.08	0.83	−0.56	0.73
	Parent C	0.00	1.00	−0.57	0.57
Prompting	Parent A	1.00	0.01 *	0.40	1.00
	Parent B	0.79	0.04 *	0.15	1.00
	Parent C	0.17	0.63	−0.40	0.74
Expanding	Parent A	0.20	0.58	−0.40	0.80
	Parent B	0.08	0.83	−0.56	0.73
	Parent C	−0.50	0.15	−1.00	0.07
Total	Parent A	0.93	0.01 *	0.33	1.00
	Parent B	0.50	0.20	−0.14	1.00
	Parent C	−0.44	0.20	−1.00	0.13

Note. LL = lower limit, UL = upper limit. * *p* < 0.05.

**Table 3:** Effect sizes for parents’ implementation of intervention strategies on children’s target communication behaviors.

Dyad	Target Communication Behavior	Tau-U	*p*-Value	Lower Limit CI 90%	Upper Limit CI 90%
Child A	Spontaneous Comments	0.73	0.04 *	0.13	1.00
Child B	Spontaneous Comments	1.00	0.00 ***	0.36	1.00
Child C	Asking Questions	1.00	0.00 ***	0.43	1.00

Note. LL = lower limit, UL = upper limit. * *p* < 0.05, *** *p* < 0.001.

## Data Availability

Data are contained within the article.

## References

[B1-behavsci-15-00118] Abdeen F. H., Albiladi W. S. (2022). Factors influencing the adoption of virtual reality (VR) technology among parents of individuals with ASD. Interactive Learning Environments.

[B2-behavsci-15-00118] Acar S., Chen C. I., Xie H. (2021). Parental involvement in developmental disabilities across three cultures: A systematic review. Research in Developmental Disabilities.

[B3-behavsci-15-00118] American Psychiatric Association (2022). Diagnostic and statistical manual of mental disorders (DSM-5-TR).

[B4-behavsci-15-00118] Antezana L., Scarpa A., Valdespino A., Albright J., Richey J. A. (2017). Rural trends in diagnosis and services for autism spectrum disorder. Frontiers in Psychology.

[B5-behavsci-15-00118] Autism Speaks (2021). Autism statistics and facts.

[B6-behavsci-15-00118] Benson S. S., Dimian A. F., Elmquist M., Simacek J., McComas J. J., Symons F. J. (2018). Coaching parents to assess and treat self-injurious behaviour via telehealth. Journal of Intellectual Disability Research.

[B7-behavsci-15-00118] Boyd B. A., McDonough S. G., Rupp B., Khan F., Bodfish J. W. (2011). Effects of a family-implemented treatment on the repetitive behaviors of children with autism. Journal of Autism and Developmental Disorders.

[B8-behavsci-15-00118] Brookman-Frazee L. (2004). Using parent/clinician partnerships in parent education programs for children with autism. Journal of Positive Behavior Interventions.

[B9-behavsci-15-00118] Brossart D. F., Laird V. C., Armstrong T. W. (2018). Interpreting Kendall’s Tau and Tau-U for single-case experimental designs. Cogent Psychology.

[B10-behavsci-15-00118] Brown J. A., Woods J. J. (2015). Effects of a triadic parent-implemented home-based communication intervention for toddlers. Journal of Early Intervention.

[B11-behavsci-15-00118] Cheng W. M., Smith T. B., Butler M., Taylor T. M., Clayton D. (2023). Effects of parent-implemented interventions on outcomes of children with autism: A meta-analysis. Journal of Autism and Developmental Disorders.

[B12-behavsci-15-00118] Christopoulou M., Drosos K., Petinou K. (2022). Recent ADVANCES of telepractice for autism spectrum disorders in speech and language pathology. Neuropsychiatric Disease and Treatment.

[B13-behavsci-15-00118] Chu C. L., Chiang C. H., Lee T. H. (2015). Caregiver-mediated joint engagement intervention for young children with autism: A pilot study of the creative movement approach to dance therapy. Formosa Journal of Mental Health.

[B14-behavsci-15-00118] Curcio F., Paccia-Cooper J. (1981). Response sets in autistic echolalia. Perceptual and Motor Skills.

[B15-behavsci-15-00118] Dogoe M. S., Banda D. R., Lock R. H. (2010). Acquisition and generalization of the picture exchange communication system behaviors across settings, persons, and stimulus classes with three students with autism. Education and Training in Autism and Developmental Disabilities.

[B16-behavsci-15-00118] Eilers H. J., Hayes S. C. (2015). Exposure and response prevention therapy with cognitive defusion exercises to reduce repetitive and restrictive behaviors displayed by children with autism spectrum disorder. Research in Autism Spectrum Disorders.

[B17-behavsci-15-00118] Flippin M., Reszka S., Watson L. R. (2010). Effectiveness of the picture exchange communication system (PECS) on communication and speech for children with autism spectrum disorders: A meta-analysis. American Journal of Speech-Language Pathology.

[B18-behavsci-15-00118] Ganz J. B., Earles-Vollrath T. L., Heath A. K., Parker R., Rispoli M. J., Duran J. (2012). A meta-analysis of single case research studies on aided augmentative and alternative communication systems with individuals with autism spectrum disorders. Journal of Autism and Developmental Disorders.

[B19-behavsci-15-00118] Ganz J. B., Morin K. L., Foster M. J., Vannest K. J., Genç Tosun D., Gregori E. V., Gerow S. L. (2017). High-technology augmentative and alternative communication for individuals with intellectual and developmental disabilities and complex communication needs: A meta-analysis. Augmentative and Alternative Communication.

[B20-behavsci-15-00118] Gao X., Drani S. (2024). Parent-implemented interventions in Chinese families of children with autism spectrum disorder. Humanities and Social Sciences Communications.

[B21-behavsci-15-00118] Garbacz S. A., McIntyre L. L., Santiago R. T. (2016). Family involvement and parent-teacher relationships for students with autism spectrum disorders. School Psychology Quarterly.

[B22-behavsci-15-00118] Garretson H. B., Fein D., Waterhouse L. (1990). Sustained attention in children with autism. Journal of Autism & Developmental Disorders.

[B23-behavsci-15-00118] Gerow S., Hagan-Burke S., Rispoli M., Gregori E., Mason R., Ninci J. (2018). A systematic review of parent-implemented functional communication training for children with ASD. Behavior Modification.

[B24-behavsci-15-00118] Goldstein S., Naglieri J., Ozonoff S. (2008). Assessment of autism.

[B25-behavsci-15-00118] Hamberger R. J., Evmenova A. S., Coogle C. G., Regan K. S. (2022). Parent coaching in natural communication opportunities through bug-in-ear technology. Topics in Early Childhood Special Education.

[B26-behavsci-15-00118] Hodgson A. R., Freeston M. H., Honey E., Rodgers J. (2017). Facing the unknown: Intolerance of uncertainty in children with autism spectrum disorder. Journal of Applied Research in Intellectual Disabilities.

[B27-behavsci-15-00118] Hong E. R., Ganz J. B., Neely L., Gerow S., Ninci J. (2016). A review of the quality of primary caregiver-implemented communication intervention research for children with ASD. Research in Autism Spectrum Disorders.

[B28-behavsci-15-00118] Hu C. C., Li Y., Zhou B. R., Liu C. X., Li C. Y., Zhang Y., Xu Q., Xu X. (2017). Reducing maternal parenting stress of children with autism spectrum disorder: Father’s involvement. Chinese Journal of Pediatrics.

[B29-behavsci-15-00118] Ingersoll B., Wainer A. (2013). Initial efficacy of Project ImPACT: A parent-mediated social communication intervention for young children with ASD. Journal of Autism and Developmental Disorders.

[B30-behavsci-15-00118] Johnson N. L., Burkett K., Reinhold J., Bultas M. W. (2016). Translating research to practice for children with autism spectrum disorder: Part 1: Definition, associated traits, prevalence, diagnostic process, and interventions. Journal of Pediatric Health Care.

[B31-behavsci-15-00118] Kaiser A. P., Hancock T. B., Nietfeld J. P. (2000). The effects of parent-implemented enhanced milieu teaching on the social communication of children who have autism. Early Education and Development.

[B32-behavsci-15-00118] Kaiser A. P., Roberts M. Y. (2013). Parent-implemented enhanced milieu teaching with preschool children with intellectual disabilities. Journal of Speech, Language, and Hearing Research.

[B33-behavsci-15-00118] Kasari C., Gulsrud A., Paparella T., Hellemann G., Berry K. (2015). Randomized comparative efficacy study of parent-mediated interventions for toddlers with autism. Journal of Consulting and Clinical Psychology.

[B34-behavsci-15-00118] Keen D., Couzens D., Muspratt S., Rodger S. (2010). The effects of a parent-focused intervention for children with a recent diagnosis of autism spectrum disorder on parenting stress and competence. Research in Autism Spectrum Disorders.

[B35-behavsci-15-00118] Kelley M. L., Heffer R. W., Gresham F. M., Elliott S. N. (1989). Development of a modified treatment evaluation inventory. Journal of Psychopathology and Behavioral Assessment.

[B36-behavsci-15-00118] Kurzrok J., McBride E., Grossman R. B. (2021). Autism-specific parenting self-efficacy: An examination of the role of parent-reported intervention involvement, satisfaction with intervention-related training, and caregiver burden. Autism.

[B37-behavsci-15-00118] Law J., Garrett Z., Nye C. (2004). The efficacy of treatment for children with developmental speech and language delay/disorder: A meta-analysis. Journal of Speech, Language, and Hearing Research.

[B38-behavsci-15-00118] Lee H.-L. (2015). The intervention strategies of social orienting and joint attention for children with autism in home setting. Special Education Quarterly.

[B39-behavsci-15-00118] Lee J. B., Cherney L. R. (2018). Tau-U: A quantitative approach for analysis of single-case experimental data in Aphasia. American Journal of Speech-Language Pathology.

[B40-behavsci-15-00118] Liang S., Zheng R. X., Zhang L. L., Liu Y. M., Ge K. J., Zhou Z. Y., Wang L. (2020). Effectiveness of parent-training program on children with autism spectrum disorder in China. International Journal of Developmental Disabilities.

[B41-behavsci-15-00118] Liao C. Y., Ganz J. B., Wattanawongwan S., Haas A., Ura S. K., Vannest K. J., Morin K. (2022). Parent coaching in a multimodal communication intervention for children with autism. Focus on Autism and Other Developmental Disabilities.

[B42-behavsci-15-00118] Lin Y. J. (2013). The study of community-based services for children with developmental delays in low resource communities. Bulletin of Special Education.

[B43-behavsci-15-00118] Liou W.-Y., Lin C.-S., Pan H.-M. (2005). The influence of caregivers’ responsive interaction on autistic children’s communicative behavior. Bulletin of Special Education and Rehabilitation.

[B44-behavsci-15-00118] Meadan H., Lee J. D., Chung M. Y. (2022). Parent-implemented interventions via telepractice in autism research: A review of social validity assessments. Current Developmental Disorders Reports.

[B45-behavsci-15-00118] Meadan H., Snodgrass M. R., Meyer L. E., Fisher K. W., Chung M. Y., Halle J. W. (2016). Internet-based parent-implemented intervention for young children with autism: A pilot study. Journal of Early Intervention.

[B46-behavsci-15-00118] National Institute of Mental Health (2024). Autism spectrum disorder. *U.S. Department of Health and Human Services, National Institutes of Health*.

[B47-behavsci-15-00118] Nevill R. E., Lecavalier L., Stratis E. A. (2018). Meta-analysis of parent-mediated interventions for young children with autism spectrum disorder. Autism.

[B48-behavsci-15-00118] Ottley J. R. (2016). Real-time coaching with bug-in-ear technology: A practical approach to support families in their child’s development. Young Exceptional Children.

[B49-behavsci-15-00118] Parker R. I., Vannest K. J., Davis J. L. (2011). Effect size in single-case research: A review of nine nonoverlap techniques. Behavior Modification.

[B50-behavsci-15-00118] Parsons M. B., Rollyson J. H., Reid D. H. (2012). Evidence-based staff training: A guide for practitioners. Behavior Analysis in Practice.

[B51-behavsci-15-00118] Peterson A. K. (2024). Across time and place: A focused review of telepractice in ASHA journals. Communication Disorders Quarterly.

[B52-behavsci-15-00118] Rankin J. A., Paisley C. A., Tomeny T. S., Eldred S. W. (2019). Fathers of youth with autism spectrum disorder: A systematic review of the impact of fathers’ involvement on youth, families, and intervention. Clinical Child and Family Psychology Review.

[B53-behavsci-15-00118] Roddy A., O’Neill C. (2019). The economic costs and its predictors for childhood autism spectrum disorders in Ireland: How is the burden distributed?. Autism.

[B54-behavsci-15-00118] Schertz H. H., Horn K., Lee M., Mitchell S. (2017). Supporting parents to help toddlers with autism risk make social connections. Young Exceptional Children.

[B55-behavsci-15-00118] Schreibman L., Dawson G., Stahmer A. C., Landa R., Rogers S. J., McGee G. G., Kasari C., Ingersoll B., Kaiser A. P., Bruinsma Y., McNerney E. (2015). Naturalistic developmental behavioral interventions: Empirically validated treatments for autism spectrum disorder. Journal of Autism and Developmental Disorders.

[B56-behavsci-15-00118] Simacek J., Dimian A. F., McComas J. J. (2017). Communication intervention for young children with severe neurodevelopmental disabilities via telehealth. Journal of Autism and Developmental Disorders.

[B57-behavsci-15-00118] Simmons C. A., Tremoulet P. D., Lecakes G. D., Williams G. J., Almon A. S., Mandayam S. (2023). User-centered development and pilot test of a virtual reality training prototype for parents of children with autism. Proceedings of the Human Factors and Ergonomics Society Annual Meeting.

[B58-behavsci-15-00118] Sparrow S. S., Cicchetti D. V., Saulnier C. A. (2016). Vineland adaptive behavior scales.

[B59-behavsci-15-00118] Syed A., Arshad U., Lovell K., Husain N. (2022). Culturally adapted parenting interventions for South Asian parents: A mixed methods systematic review. *PROSPERO 2022 CRD42022361920*.

[B60-behavsci-15-00118] Taiwan Ministry of Health and Welfare (2022). Taiwan health and welfare report.

[B61-behavsci-15-00118] Ura S., Liao C. Y., Ganz J. B., Stein K., Clerk S. (2021). Parent-coaching telehealth intervention for youth with autism spectrum disorder: A pilot program. Child & Family Behavior Therapy.

[B63-behavsci-15-00118] Vannest K. J., Parker R. I., Gonen O., Adiguzel T. (2016). Single case research: Web-based calculators for SCR analysis *(Version 2.0). Texas A&M University*.

[B62-behavsci-15-00118] van Santen J. P., Sproat R. W., Hill A. P. (2013). Quantifying repetitive speech in autism spectrum disorders and language impairment. Autism Research: Official journal of the International Society for Autism Research.

[B64-behavsci-15-00118] Vismara L. A., McCormick C., Young G. S., Nadhan A., Monlux K. (2013). Preliminary findings of a telehealth approach to parent training in autism. Journal of Autism and Developmental Disorders.

[B65-behavsci-15-00118] Wacker D. P., Berg W. K., Harding J. W., Barretto A., Rankin B., Ganzer J. (2005). Treatment effectiveness, stimulus generalization, and acceptability to parents of functional communication training. Educational Psychology.

[B66-behavsci-15-00118] Washburn J. (2012). Parent evaluation of a parent training program of positive behavior interventions.

[B67-behavsci-15-00118] Yang L. Y., Cheng P. C., Chen C. H. (2024). Effectiveness of telepractice for speech-language pathology in children with verbal communication disorders: A systemic review. The Journal of Health Sciences.

[B68-behavsci-15-00118] Yang Y., Ge X. (2015). A case study of the intervention of the family-centered positive behavior support on the screaming behavioral of children with autism. A Journal of Modern Special Education.

